# Botulinum Toxin Type A for Cervicogenic Headache: A Review of the Current Evidence

**DOI:** 10.7759/cureus.113464

**Published:** 2026-07-27

**Authors:** Catarina Vasconcelos Fonseca, Alexandre Camões-Barbosa

**Affiliations:** 1 Department of Physical Medicine and Rehabilitation, Centro Hospitalar de Trás os Montes e Alto Douro, Vila Real, PRT; 2 Clinical Neurophysiology Unit, Centro Hospitalar Universitário de Lisboa Central, Lisbon, PRT

**Keywords:** botulinum toxin type a, cervicogenic headache, pain management, secondary headache, trigger point injections

## Abstract

Cervicogenic headache (CH) is a secondary headache disorder arising from cervical structures and is frequently associated with pain, disability, and reduced quality of life. Botulinum toxin type A (BoNT-A) has been proposed as a therapeutic option due to its muscle-relaxing and antinociceptive properties; however, its efficacy in CH remains uncertain. This review aimed to evaluate the current evidence regarding the use of BoNT-A in the treatment of cervicogenic headache. A literature search was conducted in the PubMed/MEDLINE and Scopus databases, and studies assessing BoNT-A in adults with CH were included. Data on study characteristics, injection protocols, clinical outcomes, and adverse events were extracted and qualitatively synthesized. Five studies met the inclusion criteria, comprising four randomized controlled trials and one prospective study. Considerable heterogeneity was observed in diagnostic criteria, toxin formulations, injection techniques, targeted muscles, and outcome measures. Most studies reported reductions in headache intensity and frequency following BoNT-A treatment, particularly in trigger point-guided protocols and chronic or treatment-resistant populations. The trapezius and splenius capitis were the most frequently targeted muscles. Nevertheless, one high-quality placebo-controlled trial found no significant benefit over placebo. Overall, BoNT-A was well tolerated, with no serious adverse events reported. Although several studies suggest potential benefits of BoNT-A for cervicogenic headache, the current evidence remains inconclusive. Further well-designed studies using standardized diagnostic criteria and injection protocols are needed to better define its therapeutic role.

## Introduction and background

Cervicogenic headache (CH) is a clinical entity characterized by referred pain perceived in the head but originating from structures of the cervical spine, including joints, muscles, ligaments, and nerve roots of the upper cervical segments [1].

First described by Sjaastad in the 1980s, CH was later incorporated into the International Headache Society (IHS) classification and is recognized as an important cause of chronic headache and disability [1,2].

Its prevalence is estimated at 0.4-2.5% in the general population and 15-20% among patients presenting with headache [3].

The pathophysiology of CH is thought to involve convergence of nociceptive afferents from cervical and trigeminal structures within the trigeminocervical nucleus, allowing pain originating in the neck to be perceived in the head [4].

Multiple cervical structures may act as pain generators, including facet joints, intervertebral discs, and soft tissues. Increasing evidence supports a relevant role of cervical myofascial tissues in the initiation and perpetuation of headache symptoms. Indeed, stimulation of cervical myofascial trigger points can reproduce headache patterns, suggesting their contribution to the genesis or maintenance of pain [1,5].

Despite advances in understanding its mechanisms, the management of cervicogenic headache remains challenging. A wide range of therapeutic strategies have been proposed, including analgesics, nerve blocks, physical therapy, infiltrative techniques, and surgical interventions, often with variable and inconsistent outcomes. In this context, botulinum toxin type A (BoNT-A) has emerged as a potential therapeutic option [6-8].

BoNT-A is a presynaptic neurotoxin produced by *Clostridium botulinum* spp. that acts as an enzyme that reversibly cleaves the SNARE complex, inhibiting the release of acetylcholine at the neuromuscular junction, thus leading to dose-dependent muscle relaxation [9]. It has been used for medical purposes for more than thirty years now, and with an expanding array of on-label uses, including spasticity, cervical dystonia, blepharospasm, and hemifacial spasm; and off-label uses, such as jaw tremor, neuropathic pruritus, and many others [10-14].

Beyond its muscle-relaxing effects, growing evidence suggests that BoNT-A may also modulate pain pathways by inhibiting the release of neurotransmitters involved in peripheral and central sensitization [15]. These properties make it particularly appealing in conditions associated with muscular dysfunction and nociceptive sensitization, such as cervicogenic headache.

In fact, BoNT-A has been used successfully in several pain conditions, such as spasticity-associated pain, trigeminal neuralgia, temporomandibular joint disorder, central post-stroke pain, thoracic outlet syndrome, and several others [16-21].

However, the available evidence regarding the efficacy of BoNT-A in CH remains inconsistent. In a review published in 2002, only one randomized controlled trial and one case report were identified, with conflicting findings reported across studies [6].

Given these conflicting findings, the role of botulinum toxin type A in the treatment of cervicogenic headache remains unclear. Therefore, a comprehensive synthesis of the available evidence is warranted. This review aims not only to summarize the current evidence regarding the efficacy of BoNT-A for cervicogenic headache, but also to provide a practical overview of injection approaches that may inform clinical practice and future research.

## Review

Methods

Search Strategy

A comprehensive literature search was conducted using PubMed/MEDLINE and Scopus databases. The research aimed to identify studies evaluating the efficacy of BoNT-A in the treatment of cervicogenic headache.

The search strategy was structured around three main conceptual domains: (1) cervicogenic headache, (2) botulinum toxin type A, and (3) clinical outcomes related to headache (including pain intensity, frequency, and duration). Both controlled vocabulary terms (e.g., MeSH terms) and free-text keywords were used, combined with Boolean operators (AND, OR) to optimize sensitivity and specificity: (((headache disorders, secondary) OR (cervicogenic headache) OR (cervical)) AND ((Botulinum Toxins) OR (botulinum) OR (botulinum toxin) OR (toxin))) AND ((reduced headache) OR (reduced headache severity) OR (severity) OR (pain relief) OR (reduced headache days) OR (headache free days))). Database-specific filters were applied to restrict results to Clinical Trial and Randomized Controlled Trial publications in PubMed and to Article records in Scopus.

The research was performed in March 2026, with no restrictions on publication year or language. For articles published in languages other than English, eligibility was initially assessed using available English abstracts. Full texts of potentially eligible non-English articles were translated using an online machine translation tool for further evaluation.

Eligibility Criteria

Eligible studies required a clinical diagnosis of cervicogenic headache based on recognized diagnostic criteria, including those established by the IHS in the International Classification of Headache Disorders (ICHD), and the Sjaastad criteria. Only studies evaluating BoNT-A as a therapeutic intervention were included. Randomized controlled trials, controlled clinical trials, and observational studies were considered eligible, provided they reported clinical outcomes related to headache, such as pain intensity, frequency, duration, or functional impact.

Studies were excluded if they involved mixed headache populations without separate analysis for cervicogenic headache, if they did not report relevant clinical outcomes, or if they consisted solely of case reports, narrative opinions, or expert commentary without original data.

Study Selection and Data Extraction

Two reviewers independently screened titles and abstracts, assessed full-text eligibility, and resolved disagreements through discussion and consensus.

All records identified through the database search were initially screened based on titles and abstracts to assess their potential relevance. Studies that did not meet the eligibility criteria were excluded at this stage. The full texts of the remaining articles were then retrieved and independently assessed for eligibility according to the predefined inclusion and exclusion criteria. 

Particular attention was given to the diagnostic definition of cervicogenic headache and the use of botulinum toxin type A as a therapeutic intervention. When necessary, uncertainties regarding study eligibility were resolved through discussion and consensus.

Extracted information included study characteristics (such as design, sample size, and setting), patient demographics and diagnostic criteria, as well as details of the intervention, including dosage, injection sites, and administration techniques of BoNT-A. Outcome measures were also collected, focusing on clinically relevant variables such as headache intensity, frequency, duration, and functional impact, along with follow-up periods. Additionally, information regarding adverse events and safety profiles was recorded when available.

Quality Assessment

The risk of bias and quality of each included article was assessed with validated tools, according to the design of each study. Randomized clinical trials (RCTs) were assessed with the revised Cochrane Risk-of-Bias Tool for Randomized Clinical Trials (RoB2) [22], and nonrandomized observational prospective was evaluated with the MINORS tool (Methodological Index for Non-Randomized Studies) [23].

The RoB 2 tool evaluates the risk of bias in randomized controlled trials across five domains: the randomization process, deviations from intended interventions, missing outcome data, outcome measurement, and selection of the reported result. Based on these domains, each RCT is classified as low risk, some concerns, or high risk. The risk of bias of the RCTs is represented in Table 1. The MINORS tool involves assessing the methodological quality of non-randomized studies using a validated checklist. For non-comparative studies (like single-group clinical trials), only the first eight items are applicable, and each is scored as: 0) Not reported, 1) Reported but inadequate, 2) Reported and adequate. The maximum score is 16 for such studies. The study by Karadaş et al. achieved a score of 10 out of 16, indicating moderate methodological quality, with limitations mainly related to incomplete follow-up reporting, lack of blinded outcome assessment, and absence of a prospective sample size calculation [3].

**Table 1:** Risk of bias of randomized clinical trials, according to Cochrane Risk-of-Bias tool for randomized clinical trials (RoB2). +, low risk of bias; ?, some concerns; - high risk of bias. RoB2 tool: [22].

Studies	Freund et al., 2000 [24]	Zhang et al., 2003 [25]	Karadaş et al., 2011 [3]	Linde et al., 2011 [26]
Randomization process	?	?	?	+
Deviations from intended intervention	+	?	?	+
Missing outcome data	?	?	+	?
Measurement of the outcome	?	+	?	+
Selection of reported result	?	?	?	+

Data Synthesis

Given the expected heterogeneity in study design, patient populations, and outcome measures, a qualitative synthesis of the findings was performed. Results were analyzed descriptively, focusing on the overall efficacy of BoNT-A, consistency of findings across studies, and identification of potential responder subgroups.

Results

Study Designs and Baseline Characteristics

**Figure 1:**
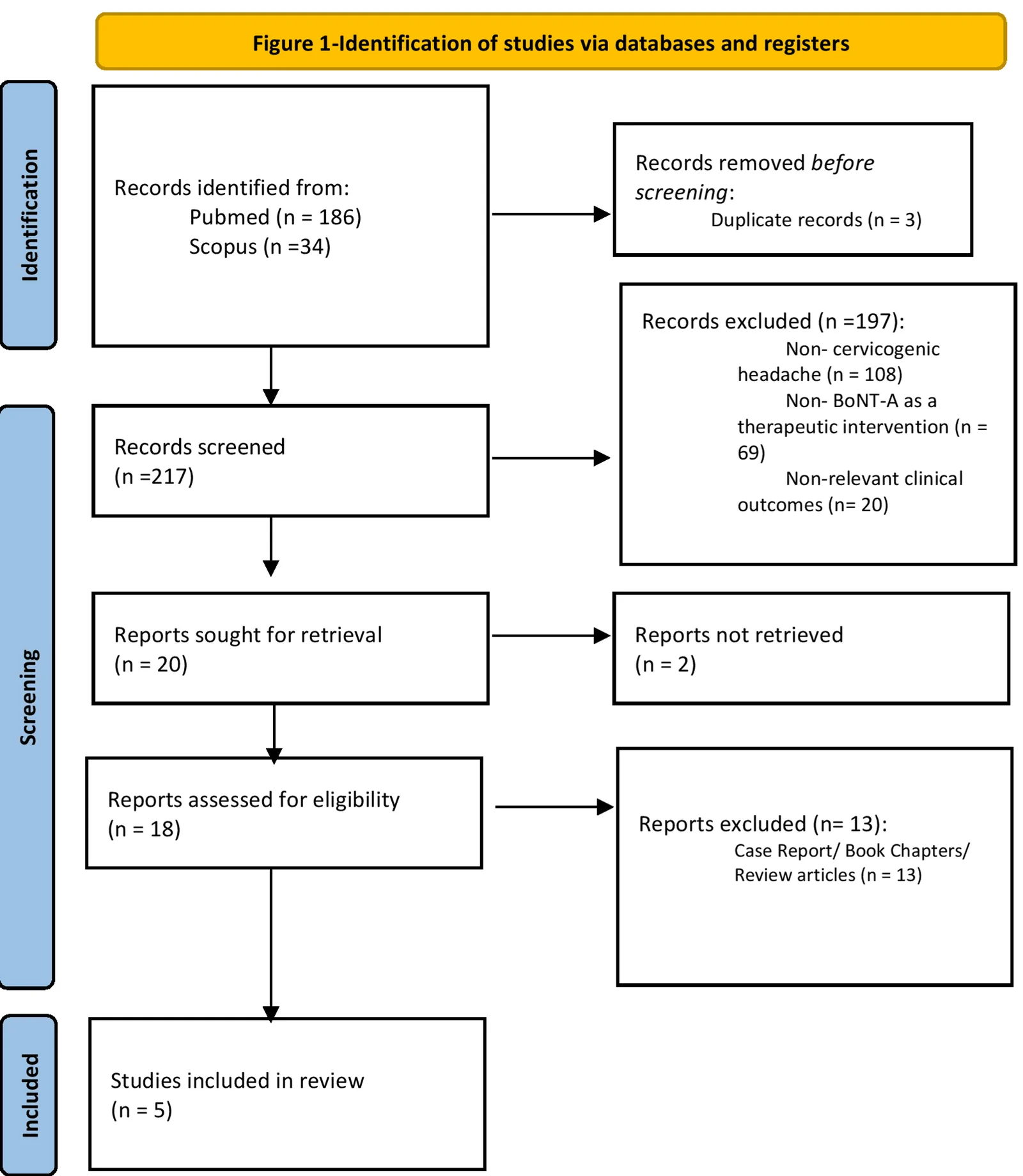
PRISMA flow diagram of study selection. PRISMA, Preferred Reporting Items for Systematic Reviews and Meta-Analyses.

The database searches identified 186 publications on PubMed and 34 articles on Scopus on the topic of botulinum toxin type A treatment of cervicogenic headache. After removal of duplicate records, the identified studies were screened based on titles and abstracts. A subset of potentially relevant articles (n=18) was selected for full-text review. Following full-text assessment, studies that did not meet the predefined eligibility criteria were excluded, most commonly due to the inclusion of mixed headache populations without separate analysis for cervicogenic headache and lack of evaluation of BoNT-A. The selection process is shown in Figure 1. 

A total of five studies were included in this review, comprising four randomized controlled trials and one prospective study. The included studies showed considerable heterogeneity in terms of study design, sample size, and methodological quality [3,24-27].

Baseline characteristics of the study populations were broadly comparable across studies, with participants typically consisting of adults with chronic cervicogenic headache, often refractory to conventional treatments. Diagnostic criteria varied between studies. Most trials used criteria consistent with those of the IHS [28] through the ICHD, although not always explicitly stated. Linde et al. applied stricter criteria based on the Cervicogenic Headache International Study Group (CHISG), including confirmation by nerve block [26]. In contrast, Freund et al. included patients with cervical-associated headache following whiplash injury, which may not be fully representative of typical cervicogenic headache populations [24].

Sample sizes were generally small, and follow-up durations ranged from two weeks to three months.

Intervention protocols varied considerably across studies. Botulinum toxin type A was administered using different doses, injection sites, and techniques, including both fixed-site approaches and injections guided by myofascial trigger points. Targeted muscles commonly included cervical and pericranial muscles such as the trapezius, splenius capitis, sternocleidomastoid, and temporal muscles, with total doses of 100 U of onabotulinumtoxinA and 150 U of abobotulinumtoxinA (fixed protocols), and up to 100 U of Lantox®. Placebo-controlled designs typically used saline injections administered at corresponding sites.

Clinical Outcomes

The clinical outcomes reported across the included studies were heterogeneous, with conflicting evidence regarding the efficacy of BoNT-A in CH.

Overall, although most included studies suggested a beneficial effect of BoNT-A on headache-related outcomes, the strength of the evidence remains limited by the small number of studies and variable methodological quality. In a randomized, double-blind, placebo-controlled trial, Zhang et al. reported a significant reduction in both the number of headache days per month and the headache index at two weeks and four weeks following injection, compared with both baseline and placebo. They injected Lantox®, 5 U per cervical trigger point, up to 100 U in total. The muscles targeted in the trigger point injections were the trapezius, the sternocleidomastoid, the splenius capitis, and the temporal muscles [25].

Similarly, Karadaş et al. observed significant reductions in pain intensity and headache frequency at six weeks and 12 weeks post-treatment in patients receiving abobotulinumtoxinA, with sustained superiority over placebo at later follow-up [3]. They injected predefined cranial and cervical muscles, but did not specify per-point dosing. The targeted muscles were the frontalis, temporalis, semispinalis capitis, splenius capitis, and the trapezius muscles. These findings support a potential therapeutic effect, particularly in patients with chronic or treatment-resistant cervicogenic headache.

Consistent with these results, the pilot randomized controlled study by Freund et al. demonstrated significant improvements in both subjective pain scores and cervical range of motion following injections of onabotulinumtoxinA up to 100 U (5 cervical trigger points, 20 U per point), in the splenius capitis, semispinalis capitis, trapezius, and “rectus capitis” [24]. No significant changes were observed in the placebo group. Although limited by small sample size and baseline group differences, these findings further suggest a beneficial effect of abobotulinumtoxinA, particularly in patients with cervical-associated headache related to musculoskeletal dysfunction [24]. The findings of Freund et al. should be interpreted cautiously, as the diagnosis of cervicogenic headache was established before the adoption of current ICHD criteria, potentially resulting in a heterogeneous study population [24].

In addition to pain-related outcomes, one study also explored broader clinical effects. Karadaş et al. (secondary analysis) reported significant reductions in headache severity and frequency following treatment, accompanied by improvements in anxiety levels, although no significant changes were observed in depression scores [27]. This suggests that BoNT-A, namely the formulation abobotulinumtoxinA that was used in this study, may have additional benefits beyond pain reduction, potentially related to improvements in quality of life [27].

In contrast, Linde et al. found no significant differences between BoNT-A and placebo in the primary outcome of reduction in moderate-to-severe headache days, nor in secondary outcomes including headache intensity, frequency, duration, or analgesic use. They injected 100 U of onabotulinumtoxinA into predefined cervical muscles (20 U per muscle), which included occipital muscle insertions, and the trapezius, splenius capitis, sternocleidomastoid, and levator scapulae muscles (fixed unilateral sites). Improvements were observed in both groups, suggesting a potential placebo effect or natural fluctuation of symptoms. Notably, no meaningful differences were found in quality-of-life measures or cervical mobility [26].

Table 2 summarizes the design and characteristics of each included study and obtained outcomes.

**Table 2:** Characteristics and clinical outcomes of the included studies. ROM, Range of movement; VAS, Visual Analogue scale; QoL, Quality of life; SF-36, Short Form-36 Health Survey; BAI, Beck Anxiety Inventory; BDI, Beck Depression Inventory.

Study	Age (mean)	Sex (F %)	Sample size	Intervention details	Injected muscles	Outcome measures	Primary findings
Freund et al., 2000 [24]	46 yrs	58%	26	100 U onabotulinumtoxinA injected into 5 cervical trigger points (20 U per trigger point) vs. placebo	Splenius capitis, rectus capitis, semispinalis capitis, trapezius (trigger point–guided)	VAS pain, cervical ROM	Significant improvement in pain and ROM at 4 weeks in onabotulinumtoxinA group; no change in placebo;
Zhang et al., 2003 [25]	42 yrs	55%	40	BoNT-A Lantox® (≤100 U) injected into multiple cervical trigger points (5U per trigger point) vs. saline solution	Trapezius, splenius capitis, sternocleidomastoid, temporal muscles (trigger point–guided)	Headache days/month, headache index	Significant reduction in headache days and headache index at 2 weeks and 4 weeks vs. baseline and placebo;
Karadaş et al., 2011 [3]	~39–40 yrs	~80%	40	150 U abobotulinumtoxinA injected into predefined cranial and cervical muscles vs. placebo (per-point distribution not specified)	Frontalis, temporalis, semispinalis capitis, splenius capitis, trapezius (fixed anatomical sites)	VAS pain, headache frequency	Significant reduction in pain intensity and frequency at 6 weeks and 12 weeks; superior to placebo at 12 weeks;
Karadaş et al., 2011 (psychological outcomes) [27]	41.9 yrs	67%	18	150 U abobotulinumtoxinA injected into predefined cranial and cervical muscles vs. placebo (10-20U per muscle)	Frontalis, temporalis, semispinalis capitis, splenius capitis, trapezius (fixed anatomical sites)	VAS pain, headache frequency, anxiety (BAI), depression (BDI)	Reduction in pain, frequency, and anxiety; no significant change in depression;
Linde et al., 2011 [26]	46.2 yrs	61%	28	100 U onabotulinumtoxinA injected into predefined neck muscle sites (20 U per muscle)	Occipital muscle insertions, trapezius, splenius capitis, sternocleidomastoid, levator scapulae (fixed unilateral anatomical sites)	Headache days/week, intensity, duration, QoL (SF-36), ROM	No significant difference between onabotulinumtoxinA and placebo in primary or secondary outcomes;

Table 3 reviews the target muscles and BoNT-A dosing parameters across included studies.

**Table 3:** Target muscles and botulinum toxin A dosing parameters across included studies. ona-, onabotulinumtoxinA; abo-,  abobotulinumtoxinA; SCM, sternocleidomastoid.

Muscle	Studies	Formulation	Dose per point	Dose per muscle
Trapezius	Freund 2000 [24]; Zhang 2003 [25]; Karadaş 2011 [3,27]; Linde 2011 [26]	ona-, abo-, Lantox®	5–20 U	15-20 U
Splenius capitis	Freund 2000 [24]; Zhang 2003 [25]; Karadaş 2011 [3, 27]; Linde 2011 [26]	ona-, abo-, Lantox®	5–20 U	15-20 U
Semispinalis capitis	Freund 2000 [24]; Karadaş 2011 [3,27]	ona-, abo-	20 U	15 U
“Rectus capitis”	Freund 2000 [24]	ona-	20 U	20 U
Sternocleidomastoid (SCM)	Zhang 2003 [25]; Linde 2011 [26]	Lantox®; ona-	5 U	20 U
Temporalis	Zhang 2003 [25]; Karadaş 2011 [3,27]	Lantox®; abo-	5 U	20 U
Frontalis	Karadaş 2011 [3,27]	abo-	Not specified	10 U
Occipital insertions	Linde 2011 [26]	ona-	10 U	20 U
Levator scapulae	Linde 2011 [26]	ona-	Not specified	20 U

Discussion

Several included studies reported reductions in headache intensity and frequency following BoNT-A administration. These findings were particularly evident in trials using trigger point-guided injection techniques and in populations with chronic or treatment-resistant symptoms. In addition, improvements in cervical range of motion and, in one study, anxiety scores were observed, suggesting a potential broader clinical impact beyond pain reduction. However, these positive findings were predominantly derived from studies with smaller sample sizes and methodological limitations, including incomplete reporting of randomization procedures, lack of standardized diagnostic criteria, and reliance on subjective outcome measures.

In contrast, the most methodologically robust trial included in this review did not demonstrate significant differences between BoNT-A and placebo across primary and secondary outcomes. This study employed a randomized, double-blind, placebo-controlled design with rigorous diagnostic criteria, standardized outcome assessment, and appropriate statistical analysis, thereby providing higher internal validity.

Recent reviews have further evaluated treatments for cervicogenic headache but differ in scope from the present review. The Cochrane review [29] concluded that evidence supporting botulinum toxin for myofascial pain syndromes was insufficient, without specifically addressing cervicogenic headache. More recently, Koonalinthip et al. performed a network meta-analysis comparing multiple treatment modalities for cervicogenic headache, identifying multimodal non-pharmacological interventions as the most effective short-term approach [30]. In contrast, the present review focuses specifically on botulinum toxin type A, providing a detailed comparison of toxin formulations, targeted muscles, and dosing strategies, in addition to summarizing the available evidence on clinical efficacy.

Several factors may contribute to the variability observed across studies. First, heterogeneity in patient selection is likely to play a relevant role. Differences in diagnostic criteria, including the use of clinically defined cervicogenic headache versus stricter criteria requiring confirmatory nerve blocks, may result in the inclusion of distinct patient populations. Second, variation in injection protocols-including total dose, anatomical targets, and use of fixed-site versus trigger point-guided techniques-may influence treatment outcomes. Third, differences in outcome measures and follow-up duration may further limit comparability between studies.

## Conclusions

Overall, the available evidence demonstrates heterogeneous results, with some studies reporting clinically meaningful improvements in headache-related outcomes, while others failed to show a significant benefit over placebo. There is considerable heterogeneity in study design, diagnostic criteria, and intervention protocols. Also, the number of available studies is limited, which can affect the strength and generalizability of the evidence.

Further well-designed randomized controlled trials, optimized injection protocols, and robust outcome measures are needed to clarify the role of BoNT-A in the management of cervicogenic headache.
